# Mitochondrial DNA Genomes Organization and Phylogenetic Relationships Analysis of Eight Anemonefishes (Pomacentridae: Amphiprioninae)

**DOI:** 10.1371/journal.pone.0123894

**Published:** 2015-04-13

**Authors:** Jianlong Li, Xiao Chen, Bin Kang, Min Liu

**Affiliations:** 1 State Key Laboratory of Marine Environmental Science, College of Ocean and Earth Sciences, Xiamen University, Xiamen, Fujian, China; 2 Guangxi Key Laboratory for Mangrove Conservation and Utilization, Guangxi Mangrove Research Center, Guangxi Academy of Sciences, Beihai, Guangxi, China; 3 Fisheries College, Jimei University, Xiamen, Fujian, China; BiK-F Biodiversity and Climate Research Center, GERMANY

## Abstract

Anemonefishes (Pomacentridae Amphiprioninae) are a group of 30 valid coral reef fish species with their phylogenetic relationships still under debate. The eight available mitogenomes of anemonefishes were used to reconstruct the molecular phylogenetic tree; six were obtained from this study (*Amphiprion clarkii*, *A*. *frenatus*, *A*. *percula*, *A*. *perideraion*, *A*. *polymnus* and *Premnas biaculeatus*) and two from GenBank (*A*. *bicinctus* and *A*. *ocellaris*). The seven *Amphiprion* species represent all four subgenera and *P*. *biaculeatus* is the only species from *Premnas*. The eight mitogenomes of anemonefishes encoded 13 protein-coding genes, two rRNA genes, 22 tRNA genes and two main non-coding regions, with the gene arrangement and translation direction basically identical to other typical vertebrate mitogenomes. Among the 13 protein-coding genes, *A*. *ocellaris* (AP006017) and *A*. *percula* (KJ174497) had the same length in ND5 with 1,866 bp, which were three nucleotides less than the other six anemonefishes. Both structures of ND5, however, could translate to amino acid successfully. Only four mitogenomes had the tandem repeats in D-loop; the tandem repeats were located in downstream after Conserved Sequence Block rather than the upstream and repeated in a simply way. The phylogenetic utility was tested with Bayesian and Maximum Likelihood methods using all 13 protein-coding genes. The results strongly supported that the subfamily Amphiprioninae was monophyletic and *P*. *biaculeatus* should be assigned to the genus *Amphiprion*. *Premnas biaculeatus* with the *percula* complex were revealed to be the ancient anemonefish species. The tree forms of ND1, COIII, ND4, Cytb, Cytb+12S rRNA, Cytb+COI and Cytb+COI+12S rRNA were similar to that 13 protein-coding genes, therefore, we suggested that the suitable single mitochondrial gene for phylogenetic analysis of anemonefishes maybe Cytb. Additional mitogenomes of anemonefishes with a combination of nuclear markers will be useful to substantiate these conclusions in future studies.

## Introduction

**Fig 1:**
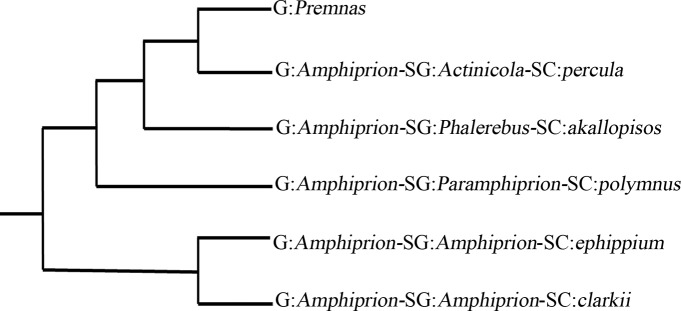
Phylogenetic relationships of the subfamily Amphiprioninae based on morphological characters (adapted from Allen [7, 8]). Abbreviations: G, Genus; SG, Subgenus; SC, Species Complex.

The subfamily Amphiprioninae (Perciformes: Pomacentridae) is a group of coral reef fishes consisting of 30 valid species, commonly known as anemonefishes because of their symbiotic association with sea anemones [1–4]. The phylogenic relationships within the Amphiprioninae are still unclear. Based on morphological characters, Allen [5, 6] suggested that the Amphiprioninae was monophyletic, and had only one genus *Amphiprion* with the two subgenera (*Amphiprion* and monotypic *Premnas*). The subgenus *Amphiprion* was further divided into five complexes, the *akallopisos* (e.g. *A*. *perideraion*), the *clarkii* (e.g. *A*. *bicinctus* and *A*. *clarkii*), the *ephippium* (e.g. *A*. *frenatus*), the *percula* (*A*. *ocellaris* and *A*. *percula*) and the *polymnus* (e.g. *A*. *polymnus*). The *clarkii* complex live with up to 10 different host anemone species and are most similar to other pomacentrids with similar deep-body shape and relatively good swimming skill, therefore, Allen [5] inferred that the *clarkii* complex maybe the most ancestral group of anemonefishes. Allen [7, 8] subsequently proposed two genera in the Amphiprioninae, *Amphiprion* and *Premnas*, and the original five complexes under the subgenus *Amphiprion* were assigned to four subgenera, i.e. *Actinicola* (the *percula* complex), *Amphiprion* (the *clarkii* and *ephippium* complexes), *Paramphiprion* (the *polymnus* complex) and *Phalerebus* (the *akallopisos* complex) (Fig 1).

While traditional classification based on morphological characters cannot provide robust phylogenetic relationships [9, 10], molecular methods are alternative tools. Generally, mitochondrial (mt) DNA genes are good molecular markers for phylogenetic studies [11]; however, short mt gene fragments exhibit limitations in resolving complicated phylogenetic relationships of many fish lineages [12]. With the development of Sanger DNA sequencing method, mitogenomes with additional informative sites have been used to resolve the deeper branches and higher-level relationships among fish taxa [13–15].

Partial mt DNA genes and nuclear genes have been used to reconstruct the phylogenetic relationships of the Amphiprioninae, and all results supported the monophyletic origin of this subfamily [16–23]. The ancestral base in the Amphiprioninae, however, is still unclear. The *percula* complex and *Premnas biaculeatus* were revealed to be the ancestral base rather than the most generalist species *A*. *clarkii* based on analyses of 12S rRNA, 16S rRNA and Cytb [16–18]. Analyses used the combination of mt genes (12S rRNA, 16S rRNA, ATP6, ATP8, COI, Cytb and ND3) and single copy nuclear genes (BMP4, RAG1 and RAG2) also inferred that the root clade of anemonefishes was the *percula* complex and *P*. *biaculeatus*, which dated back to 19 million years [19–21]. Litsios *et al*. [22] inferred that host specialist anemonefishes (the *percula* complex and *P*. *biaculeatus*) were environmental niche generalists based on seven nuclear markers (BMP4, Gylt, Hox6a, RAG1, Svep, S7 and Zic1). The most basal group was the *percula* complex only and excluded *P*. *biaculeatus* based on Cytb, 16S rRNA and the first half of mt control region [23]. To date, no studies have been conducted to elucidate the phylogenetic relationships among anemonefishes using mitogenomes.

This study utilized mitogenomes (i.e. all 13 protein-coding genes) and Bayesian and Maximum Likelihood (ML) approaches to verify the phylogenetic relationships within the Amphiprioninae. The eight mitogenomes of anemonefishes were compared; six were obtained from this study (*A*. *clarkii*, *A*. *frenatus*, *A*. *percula*, *A*. *perideraion*, *A*. *polymnus* and *P*. *biaculeatus*) and two from GenBank (*A*. *bicinctus* and *A*. *ocellaris*). The seven *Amphiprion* species represent all four subgenera even five complexes, and *P*. *biaculeatus* is the only species from *Premnas*. We tested the monophyly of the Amphiprioninae, examined the evolutionary status of *P*. *biaculeatus* within the Amphirioninae, and determined the ancestor species among anemonefishes.

## Materials and Methods

### Sample collection and identification

Specimens of six anemonefishes (*A*. *clarkii*, *A*. *frenatus*, *A*. *percula*, *A*. *perideraion*, *A*. *polymnus* and *P*. *biaculeatus*) were obtained from local aquariums (Xiamen, China) and the whole specimens were deposited in College of Ocean and Earth Sciences, Xiamen University (Table 1). After species identification [8], dorsal muscle samples were preserved in absolute ethanol solution and stored at -20°C till DNA extraction. This study was carried out in accordance with the guidelines for the Care and Use of Laboratory Animals. All anemonefishes surgery procedures were conducted under MS-222 (Tricaine Methanesulfonate) to induce sedation and anesthesia. The protocol was approved by the Animal Care and Use Ethics Committee of Xiamen University.

**Table 1:** Sampling records of six anemonefishes in this study.

Species	Subgenus	Species code	Total length (mm)	Body weight (g)
*Amphiprion percula*	*Actinicola*	APCL20130909A	42.0	2.6
*A*. *clarkii*	*Amphiprion*	AC20130831A	63.0	8.3
*A*. *frenatus*	*Amphiprion*	AF20131222A	74.0	14.1
*A*. *polymnus*	*Paramphiprion*	APLN20121207A	65.0	12.0
*A*. *perideraion*	*Phalerebus*	APRD20140328A	32.0	1.8
*Premnas biaculeatus*	—	PB20130909A	58.0	5.1

—, not applied.

### PCR amplification and sequencing

Total genomic DNA was extracted from the muscle by standard phenol-chloroform procedures [24]. The mitogenomes of anemonefishes were determined using eight consensus primer pairs with a long PCR technique (Table 2) [25]. PCR amplifications were carried out on an ABI 2700 Thermo Cycler (www.appliedbiosystems.com) in 25 μl reaction volumes, by using Takara *Ex* Taq DNA polymerase kit (www.takara.com) as indicated by the manufacturer. PCR amplifications were under the following standard cycle: one denaturation step at 94°C for 3 min, 30 cycles of 94°C for 45 s, 50~60°C for 1 min and 72°C for 1 min 30 s, followed by a final elongation step at 72°C for 10 min. PCR products were sequenced on an ABI3730XL DNA Analyzer (Sangon Biotech, www.sangon.com)

**Table 2:** Primer pairs used for mitogenomic amplification of anemonefishes.

Primer label	Upper primer sequence (5’→3’)	Primer label	Lower primer sequence (5’→3’)	Annealing temperature	Encoded genes (exclude tRNA)
131F	TTACACATGCAAGTATCCGC	2638R	TAGATAGAAACTGACCTGGATT	50°C	D-loop, 12S rRNA, 16S rRNA
2485F	ACCGAGTTACCCCAGGGAT	5468R	CACAGGTAGGATGGCTGA	50°C	ND1, ND2
5284F	TAGTTAACAGCTAAGCGC	7356R	ATTTCGATTTCTTGGGARTC	50°C	COI, COII
7106F	CCGCTCTGYCACTTTCTT	9603R	CTAGGTGATTGGAAGTCAC	50°C	COII, ATP8, ATP6, COIII
9285F	ACYTGAGCCCACCATAGCAT	11925R	TGGATTTGCACCAAGAGT	52°C	COIII, ND3, ND4L, ND4
11774F	CAAAAACATTAGATTGTGRTTC	14863R	AAGCCRCCTCARATTCATT	52°C	ND5, ND6, Cytb
13190F	CCTYAACGCCTGAGCCCT	15620R	CTGAGCTACTATTGCATCGTC	60°C	ND5, ND6, Cytb
15414F	AGGAATRCCAGTAGAACA	489R	GGGGTATCTAATCCCAGTTT	60°C	Cytb, D-loop, 12S rRNA

Standard code for mixed base sites: R = A, G; Y = C, T

### Sequence assembly and gene annotation

DNA sequences were assembled using Sequencher 4.1.4 (www.genecodes.com) to determine the mitogenomes of anemonefishes. Annotations of the mitogenomes were made by MitoAnnotator [26], including protein-coding genes, rRNA genes and non-coding regions. In addition, the tRNA genes were scanned by tRNA Scan-SE [27]. The annotation results were then submitted to NCBI by using Sequin (http://www.ncbi.nlm.nih.gov/projects/Sequin/). The organization map of mitogenomes was constructed by OrganellarGenomeDRAW [28].

### Sequence analyses

Eight mitogenomes of anemonefishes were analyzed, including the six species assembled in this study and two species available from NCBI (GenBank accession numbers were listed in Table 3). Nucleotide compositions and pairwise sequence identities for mitogenomes were calculated with BioEdit 7.1.3 [29] and MEGA 5.0 [30], respectively. For the control region, tandem repeat finder [31] was used to detect the tandem repeats. One-way ANOVA was conducted to test for significant differences in sequence variability of different regions at the level of 0.05.

**Table 3:** Sizes and nucleotide compositions for eight mitogenomes of anemonefishes.

Species	Genome size (bp)	Base compositions (%)				GenBank accession No.
		A	T	G	C	
*Amphiprion ocellaris*	16,649	29.12	25.51	15.99	29.38	AP006017*
*A*. *percula*	16,645	29.20	25.80	16.03	28.97	KJ174497
*A*. *bicinctus*	16,645	29.27	25.89	15.61	29.23	JQ030887*
*A*. *clarkia*	16,976	29.15	26.15	15.67	29.03	KJ174498
*A*. *frenatus*	16,774	29.72	25.81	15.38	29.09	KJ833752
*A*. *polymnus*	16,804	29.59	25.93	15.44	29.04	KJ101554
*A*. *perideraion*	16,579	29.37	25.50	15.68	29.45	KJ833753
*Premnas biaculeatus*	16,914	29.01	25.13	16.45	29.41	KJ833754
Mean±SD	16,748±143	29.30±0.24	25.71±0.32	15.78±0.36	29.20±0.19	—

*The mitogenome submitted by other study.—, not applied.

### Phylogenetic relationships analyses

All eight available mitogenomes of Anemonefishes (Labrodei: Pomacentridae, Amphiprioninae) and *Abudefduf vaigiensis* (Labrodei: Pomacentridae, Pomacentrinae) (GenBank accession number: AP006016) were used to phylogenetic relationships analyses. *Chaetodon auripes* (Percoidei: Chaetodontidae) (GenBank accession number: AP006004) was selected as an outgroup species.

The 10 concatenated sequences (11,445 bp) of 13 protein-coding genes were aligned by CLUSTAL X [32]. Furthermore, jModelTest 2.0 [33] was used to infer the best fitting nucleotide substitution model for the 13 protein-coding genes based on both Akaike Information Criterion correction (AICc) and Bayesian Information Criterion (BIC). The best fitting models for the 13 protein-coding genes were shown in Table 4. Phylogenetic relationships analyses of 13 protein-coding genes were performed under the Bayesian framework using MrBayes 3.2 [34]. We also tested each of the 13 protein-coding genes, 12S rRNA and the combination of Cytb+12S rRNA, COI+12S rRNA, Cytb+COI and Cytb+COI+12S rRNA with Bayesian method. Two independent analyses were run for several million generations with the Markov Chain Monte Carlo (MCMC) method each using four chains (one cold and three heated), sampling every 10 generations till the average standard deviation of split frequencies lower than 0.01 (Table 4). The first 10% of the sampled trees were discarded as “burnin”, and the remaining trees were used to obtain a 50% majority rule consensus tree with Bayesian Posterior Probability (BPP).

**Table 4:** Tree forms of different genes with best fitting nucleotide substitution model under the Bayesian framework using MrBayes.

Genes	Model	Generations	Tree form	Type
ND1	TIM2+G	2 million	(({[((1,2)3)(4,5)][(6,7)8]}9)10)	I
ND2	TPM2uf+I+G	1 million	(({[8][(((2,3)1)4)5][6,7]}9)10)	II
COI	TPM2uf+G	3 million	(({[((((2,3)1)4)5)8][6,7]}9)10)	IV
COII	TIM2+I+G	1 million	(({[(1,2,3,4)5][8][6,7]}9)10)	III
ATP8	TVM+G	4 million	({[1,2,3,4,5][((6,7)8)9]}10)	—
ATP6	TrN+I+G	1 million	(({([(((1,2)3)4)5][6,7])8}9)10)	IV
COIII	TIM2+I+G	1 million	(({[(((3,4)1)2)5][(6,7)8]}9)10)	I
ND3	TIM2+G	1 million	(({[((1,3)(2,5))4][8][6,7]}9)10)	III
ND4L	TIM2+I	1 million	(({[8][((1,3)(4)(5))2][6,7]}9)10)	II
ND4	TIM2+I+G	1 million	(({[((1,2)(3)(4))5][(6,7)8]}9)10)	I
ND5	TPM2uf+I+G	1 million	(({[(((1,2)3)4)5][(6,7)8]}9)10)	I
ND6	TPM1uf+G	1 million	(({[((1,4)(2,5)3)8][6,7]}9)10)	IV
Cytb	TIM2+I+G	3 million	(({[((((2,3)1)4)5)][(6,7)8]}9)10)	I
12S rRNA	TIM2+G	1 million	(({([(1,2,3,4)5][6,7])8}9)10)	IV
Cytb+12S rRNA	Combine the above model	1 million	(({([((1,2,3)4)5][(6,7)8]}9)10)	I
COI +12S rRNA	Combine the above model	1 million	(({[((((2,3)1)4)5)8][6,7]}9)10)	IV
COI+Cytb	Combine the above model	1 million	(({[(((2,3)1)4)5][(6,7)8]}9)10)	I
COI+Cytb+12S rRNA	Combine the above model	1 million	(({[(((2,3)1)4)5][(6,7)8]}9)10)	I
13 protein-coding genes	Combine the above model	1 million	(({[(((1,2)3)4)5][(6,7)8]}9)10)	I

The labels for the taxa used in the tree forms are 1 = *Amphiprion bicinctus*, 2 = *A*. *polymnus*, 3 = *A*. *frenatus*, 4 = *A*. *perideraion*, 5 = *A*. *clarkii*, 6 = *A*. *ocellaris*, 7 = *A*. *percula*, 8 = *Premnas biaculeatus*, 9 = *Abudefduf vaigiensis* and 10 = *Chaetodon auripes*. The tree forms can be classified four types based on the position of *P*. *biaculeatus*, which are type I (({[1~5][(6,7)8]}9)10) (i.e. the *percula* complex and *P*. *biaculeatus* were grouped into one clade, which formed the ancestral taxon of the anemonefishes), type II (({[8][1~5][6,7]}9)10) (i.e. the basal group of anemonefishes was the *percula* complex, while the *P*. *biaculeatus* was at the end of Amphiprioninae), type III (({[(1~5][8][6,7]}9)10) (i.e. the basal group of anemonefishes was the *percula* complex, while the *P*. *biaculeatus* was in the middle of Amphiprioninae) and type IV (({[(1~5)8][6,7]}9)10) (i.e. the basal group of anemonefishes was the *percula* complex, while the *P*. *biaculeatus* was the root of another clade grouped with subgenera *Amphiprion*, *Paramphiprion* and *Phalerebus*).—, not applied.

Phylogenetic relationships of 13 protein-coding genes were also inferred under the ML criterion using TREEFINDER [35]. Each analysis ran with 1,000 replicates using a random starting tree with propose TVM+G model based on AICc. Search replicates were marked by the log likelihood (lnL) scores, and only that with the best score was retained. The 50% majority consensus topology with bootstrap values was evaluated with 1,000 boot strap replications. The ML topology hypothesis was also tested under Approximately Unbiased (AU) test [36].

## Results and Discussion

### mtDNA organization and composition

**Fig 2:**
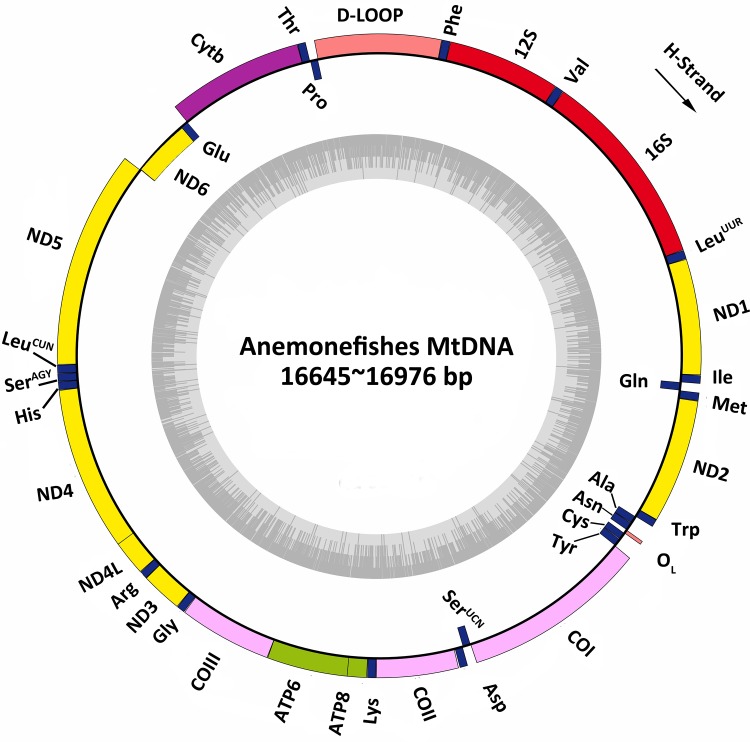
Gene organization of mitogenome in anemonefishes. Arrow indicated the orientation on H-strand (clockwise). The circle inside the GC content graph marked the 50% threshold.

The eight mitogenomes of anemonefishes encoded 13 protein-coding genes, two rRNA genes, 22 tRNA genes and two main non-coding regions, with the gene arrangement and translation direction basically identical to other typical vertebrate mitogenomes (Fig 2) [37]. The lengths of eight mitogenomes varied between 16,645 and 16,976 bp (Table 3). The overall nucleotide similarity between the eight mitogenomes was high (91.12±2.9%). In the all 37 identified genes, most genes were encoded on the heavy-strand (H-strand) with the exceptions of the ND6 and eight tRNA genes which were located on the light-strand (L-strand) (Table 5). The overall H-strand nucleotide compositions of eight mitogenomes were 29.30±0.24% A, 25.71±0.32% T, 15.78±0.36% G and 29.20±0.19% C, showed an anti-G bias (p<0.05) (Table 3).

**Table 5:** Mitogenomic genes of eight anemonefishes.

Gene or region	Nucleotide size (bp)	Amino acid size(bp)	Star codon	Stop codon^a^	Anti-codon	Intergenic nucleotides^b^	Strand
tRNA-Phe	69				GAA	0	H
12S rRNA	948~951					0	H
tRNA-Val	72~73				TAC	0	H
16S rRNA	1,695~1,698					0	H
tRNA-Leu^(UUR)^	74				TAA	0	H
ND1	975	324	ATG	TAA/TAG		0	H
tRNA-Ile	68~70				GAT	4	H
tRNA-Gln	71				TTG	-1	L
tRNA-Met	69				CAT	-1	H
ND2	1,045	348	ATG	T-		0	H
tRNA-Trp	72				TCA	0	H
tRNA-Ala	69~70				TGC	3	L
tRNA-Asn	73				GTT	1	L
O_L_	31~34					0	—
tRNA-Cys	66~67				GCA	0	L
tRNA-Tyr	71				GTA	0~1	L
COI	1,566	522	GTG	TAA		1	H
tRNA-Ser^(UCN)^	71				TGA	23~26	L
tRNA-Asp	72~73				GTC	3	H
COII	691	230	ATG	T-		1~7	H
tRNA-Lys	74				TTT	0	H
ATP8	168	55	ATG	TAA		2	H
ATP6	683	227	GTG/ATG/CTG	TA-		-10	H
COIII	785	261	ATG	TA-		0	H
tRNA-Gly	72				TCC	0	H
ND3	349	116	ATG	T-		0	H
tRNA-Arg	69				TCG	0	H
ND4L	297	98	ATG	TAA		0	H
ND4	1,381	460	ATG	T-		-7	H
tRNA-His	69				GTG	0	H
tRNA-Ser^(AGY)^	68~69				GCT	0	H
tRNA-Leu^(CUN)^	73				TAG	8~10	H
ND5	1,866~1,869	622~623	ATG	AGA		0	H
ND6	522	173	ATG	TAA/TAG		-5	L
tRNA-Glu	69				TTC	1	L
Cytb	1,141	380	ATG	T-		2	H
tRNA-Thr	72				TGT	0	H
tRNA-Pro	70~71				TGG	0~1	L
D-loop	836~1,231					0	—

^a^T—and TA- stand for incomplete codon

^b^Negative numbers indicate overlapping nucleotides between adjacent genes;—, not applied.

The cumulative lengths of the 13 protein-coding genes ranged from 11,447 to 11,451 bp, accounted for 67.4% to 68.8% of the mitogenomes. The length of ND5 was 1,866 bp in *A*. *ocellaris* and *A*. *percula*, and 1,869 bp in the rest six mitogenomes. The phenomenon was attributed to the three nucleotides deletion in the upstream (30 nucleotides) of ND5, however, both structures could translate to amino acids successfully. Protein-coding genes of the eight mitogenomes were mostly initiated by the typical start codon ATG (Table 5). There were exceptions; the COI began with GTG, which was identical to most fish mitogenomes [38, 39], as well as to chicken [40]. Additionally, except *A*. *percula* and *A*. *frenatus* which the initiation codons in ATP6 were ATG and CTG, respectively, the rest six mitogenomes retained GTG. Among the mt genetic code of vertebrates, the CTG is not a typical initiation codon; however, it was a common character in groupers (Epinephelidae) [41, 42]. Meanwhile, for the termination codons (Table 5), COI, ATP8, ND4L, ND1 and ND6 were terminated with TAA or TAG; ND5 was terminated with AGA; and the other seven protein-coding genes were stop by incomplete codon T or TA that might be able to form complete termination signal UAA via post-transcriptional polyadenylation [43]. The usage of AGA stop codon in ND5 presumably was created from the ancestral TAG stop codon by deletion of the first nucleotide T and by use of R (A or G) as the third nucleotide, which occurred very early in the evolution of metazoans [44, 45].

Among the two rRNA genes, the 12S rRNA was located between tRNA-Phe and tRNA-Val ranging from 948 to 951 bp in length and the 16S rRNA was located between tRNA-Val and tRNA-Leu^(UUR)^ ranging from 1,695 to 1,698 bp in length (Table 5). The 22 tRNA genes ranged from 66 to 74 bp in size (Table 5). Except tRNA-Ser^(AGY)^ due to lack of the entire dihydrouridine (DHU) arm [46], the remaining 21 tRNAs could be folded into the typical clover-leaf secondary structure as determined by the tRNA-scan SE program [27].

**Fig 3:**
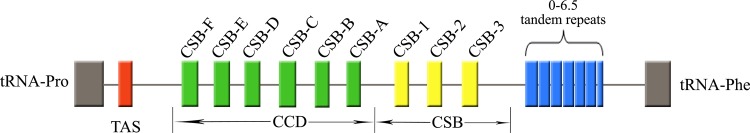
Schematic structures of mtDNA D-loops of anemonefishes. Abbreviations: TAS, Termination Associated Sequence; CCD, Central Conserved Domain; CSB, Conserved Sequence Block. CSB-F, GTA-TAAGAACCGACCAT; CSB-E, GACAA—ATTGTG-GGGT; CSB-D, TATTCCTGGCATTTGGTTCCTA-TTCAGG—CA; CSB-C, CTT-CC—————-ATAAGTTAATG; CSB-B, CAT—-ACTC-TTACCCAC; CSB-A, TC———GGTCAG—-TT; CSB-1, CATAA-TGATATCAAGAGCAT—-A; CSB-2, TAA-CCCCCCC—-CCCCCC; CSB-3, T—AAACCCCCC-G-AA-CA.

The two main non-coding regions in the eight mitogenomes were origin of L-strand replication (O_L_) and control region (D-loop) (Table 5). The O_L_ was located in the WANCY cluster [41] and varied from 31 to 34 bp in length (Table 5; Fig 2). D-loop was located between tRNA-Pro and tRNA-Phe, which ranged in size from 836 to 1,231 bp (Tables 5 and 6). The overall nucleotide compositions of D-loop were 35.86±1.46% A, 30.52±1.07% T, 13.39±1.14% G and 20.23±1.50% C (Table 6), and the AT content (66.39±2.16%) was higher than the mitogenomes (55.02±0.47%) (Table 3). The D-loop in anemonefishes consisted of three parts which were Termination Associated Sequence (TAS), Central Conserved Domain (CCD) and Conserved Sequence Block (CSB) (Fig 3). The TAS sequence was TA(G)CATATATGTA which contained the conserved TAS motif TA(G)CAT and the reversed complement TAS (cTAS) motif ATGTA. The TAS motif can pair with the cTAS motif to form a stable hairpin loop which presumably plays a significant role as sequence-specific signal for termination of mtDNA replication [47, 48]. Six CSBs (CSB-F to CSB-A) were identified in the CCD. In addition, three CSBs (CSB-1 to CSB-3) were determined after the CCD with the exception of *P*. *biaculeatus* which only had the CSB-3. In the downstream of D-loop, variable tandem repeats after the CSB were found in *A*. *clarkii*, *A*. *frenatus*, *A*. *polymnus* and *P*. *biaculeatus* (Table 6). Similar to the gobiids (Gobiidae), the tandem repeats in the downstream of D-loop were just the short fragments and repeated in a simply way [49]. In contract to the tandem repeats in the upstream of D-loop of other fishes, each tandem repeats contained a conserved TAS motif and cTAS which formed the Extended Termination Associated Sequence (ETAS) [42, 50].

**Table 6:** Sizes and nucleotide compositions for D-loop of eight anemonefishes.

Species	Size (bp)	Base compositions (%)					Tandem repeats
		A	T	G	C	A+T	
*Amphiprion ocellaris*	917	36.32	29.55	13.41	20.72	65.87	0
*A*. *percula*	910	36.70	30.11	14.07	19.12	66.81	0
*A*. *bicinctus*	900	34.89	30.22	13.33	21.56	65.11	0
*A*. *clarkia*	1,231	33.47	30.95	13.32	22.26	64.42	5.5
*A*. *frenatus*	1,030	38.54	32.72	11.07	17.67	71.26	6.5
*A*. *polymnus*	1,060	35.85	30.75	12.74	20.66	66.60	3.5
*A*. *perideraion*	836	35.53	29.19	14.47	20.81	64.72	0
*Premnas biaculeatus*	1,166	35.59	30.70	14.67	19.04	66.29	5.5
Mean±SD	1,006±140	35.86±1.46	30.52±1.07	13.39±1.14	20.23±1.50	66.39±2.16	—

—, not applied.

### Phylogenetic relationships

The topologies of each 13 protein-coding genes, 12S rRNA and four combinations (Cytb+12S rRNA, COI+12S rRNA, Cytb+COI and Cytb+COI+12S rRNA) were summarized in Table 4. Only the tree form of ATP8 did not support the Amphiprioninae as a monophyletic group; therefore, ATP8 maybe not a suitable single mt gene for phylogenetic relationships analysis of anemonefishes. The tree forms of other genes were classified four types based on the position of *P*. *biaculeatus*, which were type I (({[1~5][(6,7)8]}9)10) (i.e. the *percula* complex and *P*. *biaculeatus* were grouped into one clade, which formed the ancestral taxon of the anemonefishes), type II (({[8][1~5][6,7]}9)10) (i.e. the basal group of anemonefishes was the *percula* complex, while the *P*. *biaculeatus* was at the end of the Amphiprioninae), type III (({[(1~5][8][6,7]}9)10) (i.e. the basal group of anemonefishes was the *percula* complex, while the *P*. *biaculeatus* was in the middle of the Amphiprioninae) and type IV (({[(1~5)8][6,7]}9)10) (i.e. the basal group of anemonefishes was the *percula* complex, while the *P*. *biaculeatus* was the root of another clade grouped with subgenera *Amphiprion*, *Paramphiprion* and *Phalerebus*). Because the tree forms of ND1, COIII, ND4, Cytb, Cytb+12S rRNA, Cytb+COI and Cytb+COI+12S rRNA were similar to that 13 protein-coding genes defined as type I, we suggested that the suitable single mt gene for phylogenetic analysis of anemonefishes maybe Cytb. The tree forms constructed by Elliott *et al*. [16], Tang [17], Jang-Liaw *et al*. [18], Quenouille *et al*. [19], Cooper *et al*. [20], Litsios *et al*. [21] and Litsios *et al*. [22] were similar to type I, however, the topology in Santini and Polacco [23] was close to type IV.

**Fig 4:**
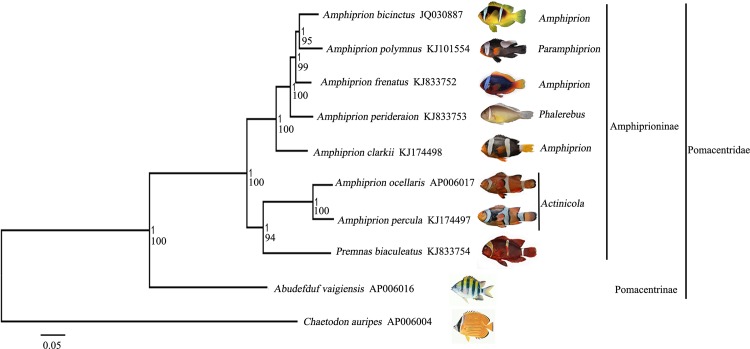
Molecular phylogenetic tree of eight anemonefishes (Amphiprioninae) and *Abudefduf vaigiensis* (Pomacentrinae) from the same family Pomacentridae in suborder Labrodei. *Chaetodon auripes* (Percoidei: Chaetodontidae) was selected as an outgroup species. Congruent tree topology was inferred from partitioned Bayesian and Maximum Likelihood analyses using the concatenated nucleotide sequences of 13 protein-coding genes. The Bayesian posterior probability values (top) and boots trap values (bottom) were labeled at branch nodes. Branch length information from the Bayesian tree was shown. GenBank accession number of each species was listed on the right of the species name.

The tree topology of 13 protein-coding genes was consistent with both the Bayesian and ML approaches of phylogenetic analyses, and the support values were robust which were above 80% bootstrap value on the ML tree and BBP of 1 on the Bayesian tree (Fig 4). The ML topology hypothesis was tested under AU test (p = 0.24), which indicated the reality of the tree. Firstly, the phylogenetic analyses topology which defined as type I supported the monophyly of Amphiprioninae (Table 4; Fig 4), same as previous studies based on partial mt DNA genes and nuclear genes [16–23]. Secondly, the genus *Amphiprion* was not a monophyletic group (Fig 4). On one hand, the *percula* complex and *P*. *biaculeatus* were grouped into one clade, which formed the ancestral taxon of the anemonefishes, as documented in previous studies [16–22]. This was in contrast to the finding of Santini and Polacco [23] who reported the basal group of anemonefishes was the *percula* complex only by using the first half sequence of the mt control region which evolves with a rapid evolutionary rate, which may lead to a reduction of resolution in the phylogenetic relationships analyses. On the other hand, the subgenera *Amphiprion*, *Paramphiprion* and *Phalerebus* formed the other clade and the basal was *A*. *clarkii*. Thirdly, the subgenus *Amphiprion* (represented by *A*.*clarkii*, *A*. *frenatus* and *A*. *bicinctus*) was not monophyletic as found in the previous studies [16–23].

## Conclusions

The eight mitogenomes of anemonefishes were compared including six newly sequenced species from this study. The eight mitogenomes encoded 13 protein-coding genes, two rRNA genes, 22 tRNA genes and two main non-coding regions. In the 13 protein-coding genes, *A*. *ocellaris* (AP006017) and *A*. *percula* (KJ174497) had the same length in ND5 with 1,866 bp, which were three nucleotides less than the other six anemonefishes. Both structures of ND5, however, could translate to amino acid successfully. Only four mitogenomes (*A*. *clarkii*, *A*. *frenatus*, *A*. *polymnus* and *P*. *biaculeatus*) of anemonefishes had the tandem repeats in D-loop; the tandem repeats were located in downstream after CSB rather than the upstream and repeated in a simply way. Applying the 13 protein-coding genes to test the suggested taxonomic reorganization of the anemonefishes, the results supported the monophyly of the subfamily Amphiprioninae and the *percula* complex together with *P*. *biaculeatus* as the ancestral taxon of the anemonefishes. The tree forms of ND1, COIII, ND4, Cytb, Cytb+12S rRNA, Cytb+COI and Cytb+COI+12S rRNA were similar to that 13 protein-coding genes, therefore, we inferred that the suitable single mt gene for phylogenetic relationships analysis of anemonefishes maybe Cytb. In addition to offer insight into the evolution of the anemonefishes, the results of this work provided important molecular resources for the further studies of identification, conservation genetics, and other phylogenetic evolution of anemonefishes.
